# The capacity to learn new motor and perceptual calibrations develops concurrently in childhood

**DOI:** 10.1038/s41598-019-45074-6

**Published:** 2019-06-27

**Authors:** Cristina Rossi, Connie W. Chau, Kristan A. Leech, Matthew A. Statton, Anthony J. Gonzalez, Amy J. Bastian

**Affiliations:** 1 Center for Movement Studies, Kennedy Krieger Institute, Baltimore, MD USA; 2 Department of Biomedical Engineering, The Johns Hopkins University School of Medicine, Baltimore, MD USA; 3 Department of Physical Therapy, Nazareth College, Rochester, NY USA; 4 Department of Neuroscience, The Johns Hopkins University School of Medicine, Baltimore, MD USA

**Keywords:** Motor control, Sensorimotor processing, Development of the nervous system, Neurophysiology

## Abstract

Learning new movements through an error-based process called motor adaptation is thought to involve multiple mechanisms which are still largely not understood. Previous studies have shown that young children adapt movement more slowly than adults, perhaps supporting the involvement of distinct neural circuits that come online at different stages of development. Recent studies in adults have shown that in addition to recalibrating a movement, motor adaptation also leads to changes in the perception of that movement. However, we do not yet understand the relationship between the processes that underlie motor and perceptual recalibration. Here we studied motor and perceptual recalibration with split-belt walking adaptation in adults and children aged 6–8 years. Consistent with previous work, we found that this group of children adapted their walking patterns more slowly than adults, though individual children ranged from slow to adult-like in their adaptation rates. Perceptual recalibration was also reduced in the same group of children compared to adults, with individual children ranging from having no recalibration to having adult–like recalibration. In sum, faster motor adaptation and the ability to recalibrate movement perception both come online within a similar age-range, raising the possibility that the same sensorimotor mechanisms underlie these processes.

## Introduction

Motor learning is not a unitary process^1–5^. During natural movement, several distinct learning mechanisms are used together and to varying degree, depending on the situation^6^. Adaptation is one form of motor learning that, driven by error, adjusts or improves a movement in the face of novel, but predictable demands^7,8^. Adaptation usually occurs on a time scale of minutes, and requires the movement to be repeated many times so that the errors experienced on one movement can be partially corrected on the next. When the novel demand is removed post-adaptation, the movement must again be practiced in order to de-adapt and return it to normal. We do not fully understand the neural mechanisms that underlie adaptation. The prevailing view is that it depends, at least in part, on the integrity of the cerebellum, as evidenced by poor learning in people with cerebellar damage^9–11^ as well as studies in animal models^12,13^. Additionally, although historically considered a single process, recent evidence suggests that motor adaptation might involve more than one mechanism and thus depend on multiple neural circuits^10,14–16^. For example, studies exploring the development of motor learning in childhood suggest that distinct adaptive mechanisms mature at different ages, supporting the involvement of multiple brain circuits in adaptation^17–20^.

Recent studies of adults have shown that motor adaptation not only recalibrates movement, but also leads to changes in movement perception. For instance, adaptation of reaching movements can alter the perception of hand position such that participants sense their hand to be in a different location than it really is^21,22^. Similarly, walking adaptation on a split-belt treadmill – where the two legs move at different speeds – alters perception of leg speed such that the leg moving ‘fast’ during adaptation begins to feel slower than it actually is^23,24^. This phenomenon is referred to as ‘perceptual recalibration’. Recent studies in both the upper and lower extremities have shown that this recalibration of movement perception with adaptation is disrupted in people with cerebellar damage^25–27^. Based on these findings, ‘t Hart and Henriques 2016 suggested that the recalibration of both movement and movement perception during adaptation could result from the same cerebellum-based trial-by-trial modification of the expected sensory outcomes of our movements^28^. Despite the growing interest in the topic, much is still unknown about the neural mechanisms involved in motor adaptation and perceptual recalibration, and about the relationship between these two phenomena.

An interesting approach to understanding the neural mechanisms involved in motor adaptation is to investigate the development of this behavior during childhood. Several studies have shown that children are slower than adults at modifying reaching, walking and eye movements through adaptive learning^18–20,29,30^. Specifically, previous work in split-belt treadmill adaptation has shown that the time-course of motor adaptation gradually changes throughout development^20^. Children aged 3–5 years do not always adapt completely, but when they do their time-course of learning tends to be slow. When children are 6–8 years old, they reliably adapt but still show, on average, an immature time-course of learning compared to adults^20^. Children older than 9 years produce an adult-like adaptation pattern. Thus, the 6–8 years old age range may be a particularly critical point in the development of brain regions associated with adaptive learning processes. This is consistent with the ongoing development of sensorimotor areas of the brain^31,32^, including the cerebellum, which reaches peak volume only after age 11^33^.

While this evidence elucidates the development of adaptive processes in the motor domain, we do not yet know if young children recalibrate their perception of movement during adaptation, and how the maturation of the perceptual recalibration in childhood relates to the rate of motor learning. Given the characteristic development of the time-course of motor adaptation between 6–8 years of age, we wondered: to what extent do children in this age window recalibrate their perception of movement with motor adaptation? We specifically investigated the recalibration of leg speed perception in children ages 6–8 years old, compared to adults, after walking on a split-belt treadmill. We hypothesized that the same mechanism underlying faster motor adaptation that is coming online in this age range may also be involved in perceptual recalibration, and hence that children would not only adapt their gait pattern slower, but also recalibrate leg speed perception less than adults.

## Results

**Figure 1:**
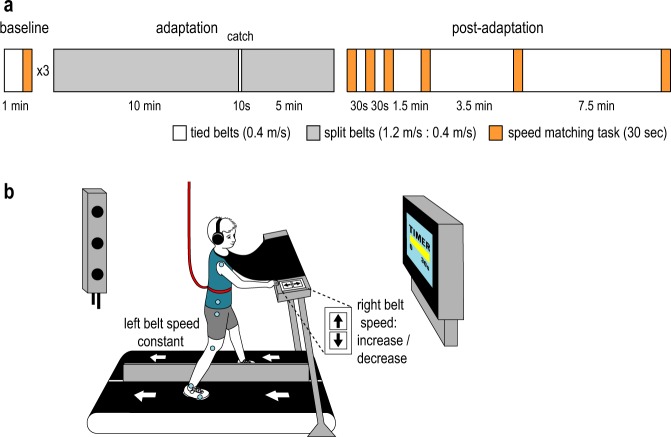
(**a**) Experimental paradigm. Blocks of tied-belt walking are depicted in white (both belts at 0.4 m/s), and blocks of split-belt walking are depicted in gray (right belt at 1.2 m/s and left belt at 0.4 m/s). Each orange block represents one iteration of the speed matching task. (**b**) Experimental setup of the speed matching tasks. The left belt moved at a constant speed of 0.4 m/s, and the participants used a two-button keypad to increase or decrease the speed of the right belt with the goal of making it match the speed of the left. A time bar on the screen informed participants of the remaining time to complete each 30-second task iteration. Participants wore a safety harness, and a cloth drape and headphones to cancel out visual and auditory cues of the treadmill belts. Kinematic data was recorded throughout the experiment by two cameras, and active markers were placed on the participant as shown.

We investigated motor adaptation and perceptual recalibration in a group of twenty-two adults (age range 18–29 years old, 12 females) and a group of twenty-two children (age range 6–8 years old, 9 females). Participants first walked on a split-belt treadmill with the belts moving at the same speed (baseline, Fig. 1a). Then, participants adapted to a 1.2:0.4 m/s split-belt condition, where the right belt was driven to move three times faster than the left. The belts were again tied at the same speed during post-adaptation in order to measure participants’ motor aftereffect. Similar to previous studies^24,25^, multiple iterations of a speed matching task were performed at specific time points during the experiment to measure participants’ leg speed perception in baseline and following adaptation (Fig. 1b).

### Adaptation of step length asymmetry

**Figure 2:**
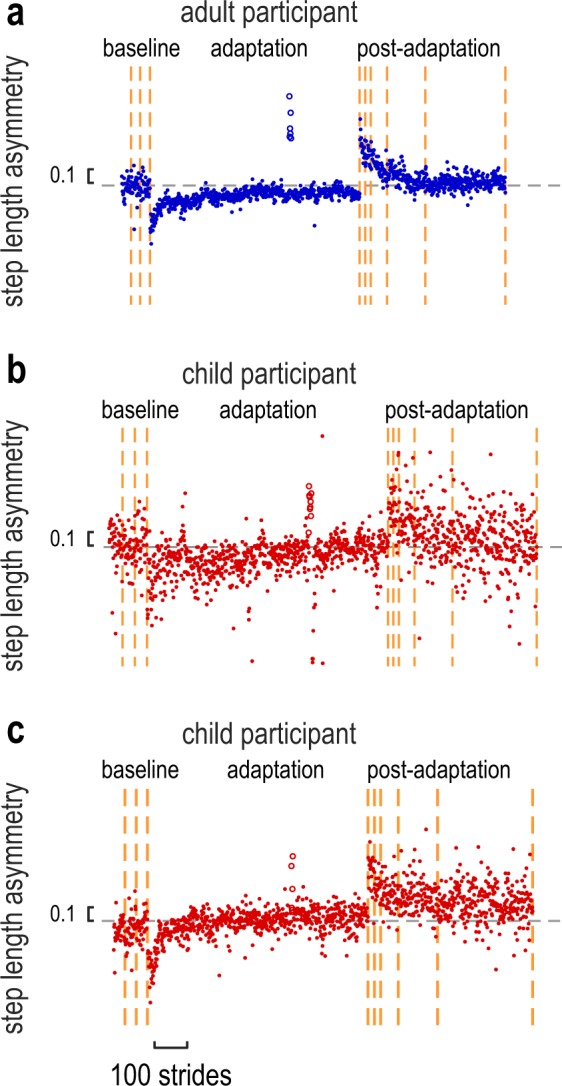
Example step length asymmetry data for an adult (**a**) and two child participants (**b,c**). Each dot depicts the step length asymmetry for a single stride; open dots depict the step length asymmetry during the catch trial. The dotted gray line represents a step length asymmetry of zero, and iterations of the speed matching tasks are indicated by the vertical dashed orange lines. Motor adaptation appeared slower and more linear for the child participant depicted in (**b**), while the child participant depicted in (**c**) adapted similar to the adult participant.

Figure 2 shows individual data that are representative of typical motor behavior for an adult and two child participants. These participants were chosen to provide intuitive examples of the different motor behaviors that we found in our dataset, which will be explained below. Step length asymmetry was used as our measure for motor learning, as it has been shown to adapt robustly to split-belt perturbations^4^. It is defined as the normalized difference between the length of the steps taken on the fast and slow belts (see Methods). As expected, the adult and both child participants walked symmetrically during baseline. Importantly, all three participants were perturbed when first exposed to split-belts and appeared to correct their asymmetry by the end of adaptation. However, the child in panel b was more variable than the adult participant and did not show the rapid correction in the first ~30 steps of the adaptation phase. The child participant depicted in panel c was, instead, more similar to the adult participant in terms of motor variability and time-course of adaptation. Participants in both groups showed aftereffects in step length asymmetry during the catch trial, as well as at the start of the post-adaptation phase. These example data indicate that participants in both the adult and child groups were able to adapt and learn to walk symmetrically within the split-belt environment, however some children adapted at a more linear rate than adults.

**Figure 3:**
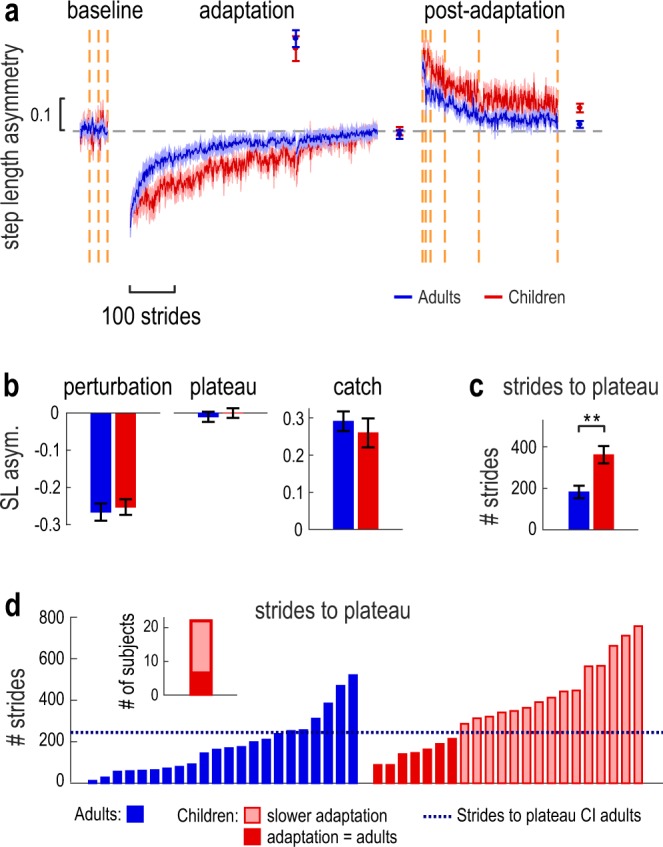
(**a**) Group step length asymmetry (mean ± SE) for the adult group (blue) and child group (red). For each paradigm block, data is truncated to the least number of strides across participants. Error bars (mean ± SE) are shown for the following epochs: catch trial (average of first 3 strides), adaptation plateau (average of last 30 strides) and post-adaptation plateau (average of last 30 strides). The vertical orange lines indicate the iterations of the speed matching task; the dotted gray line represents the step length asymmetry of zero. (**b**) Comparison of the step length asymmetry between children and adults at the initial perturbation, adaptation plateau, and catch trial (error bars represent group mean ± SE). There was no significant difference between the groups at these time epochs. (**c**) Number of strides to reach the adaptation plateau (group mean ± SE; see Methods). Children took more strides than adults to reach plateau; significant difference between the groups is indicated by **(p < 0.01). (**d**) Number of strides to plateau for each participant (blue: adults, red: children). For the child participants, solid red bars represent participants who adapted at a rate comparable to the adult group, while shaded red bars represent participants who adapted slower (based on the 95% stride to plateau CI, depicted by the dotted blue line). The inset of panel (**d**) summarizes the number of children which adapted similar to (red area) or slower than (shaded area) adults.

Figure 3a depicts the average of group step length asymmetry during baseline, adaptation and post-adaptation periods of the experiment. Baseline step length asymmetry values were not different between the two groups (t_(42)_ = −0.604, p = 0.549). During adaptation, children clearly adapted more slowly, but to the same extent as the adults. Children also appeared to de-adapt more slowly in the post-adaptation period. To compare motor adaptation between child and adult participants, we performed a repeated-measure ANOVA with factors Group and Epoch (initial adaptation perturbation, adaptation plateau, and catch trial). Figure 3b shows bar graphs representing each of these epochs. We found a significant effect of Epoch (F_(2,84)_ = 235.227, p < 0.001), however no effect of Group (p = 0.900) or Group x Epoch interaction (p = 0.561). These results indicate that both groups were equally asymmetric initially and adapted a similar amount to the split-belt treadmill. We then quantified the rate of adaptation by comparing the number of strides it took each group to reach plateau (Fig. 3c)^34^. Independent-samples t-test revealed that, although both groups adapted a similar amount, the rate of adaptation in children was significantly slower than adults (strides to plateau: 361.41 ± 42.00 vs. 182.14 ± 29.98; t_(42)_ = −3.475, p = 0.0012).

As we had observed that the time-course of adaptation differs across participants in the child group (Fig. 2), we next investigated the rate of adaptation of individual participants. Figure 3d depicts the stride to plateau values for all participants. Solid red bars represent child participants who took a similar number of strides to adults to reach their plateau step length asymmetry (based on the adult 95% CI, see Methods), while shaded red bars represent child participants who took more strides. We found that, although children adapted slower than adults on average, 7 of the 22 child participants adapted to a rate that was comparable to adults (Fig. 3d and inset). Hence, it appears that in the 6–8 years age range, children are transitioning from adapting slower to adapting at a similar rate to adults.

### Recalibration of leg speed perception

We were particularly interested in assessing perceptual recalibration following adaptation in children compared to adults. To assess changes in leg speed perception, we used a modified version of the speed matching task developed and used by Vazquez *et al*.^24^ (Fig. 1b). Each iteration of the task began with the left treadmill belt moving at 0.4 m/s and the right belt not moving. Participants were asked to press up or down keys on a keypad to adjust the speed of the right belt to match that of the left belt. As we expected leg speed perception to change after split-belt adaptation^24^, all participants performed the leg speed perception task three times during baseline, and six times during post-adaptation (see Fig. 1a).

**Figure 4:**
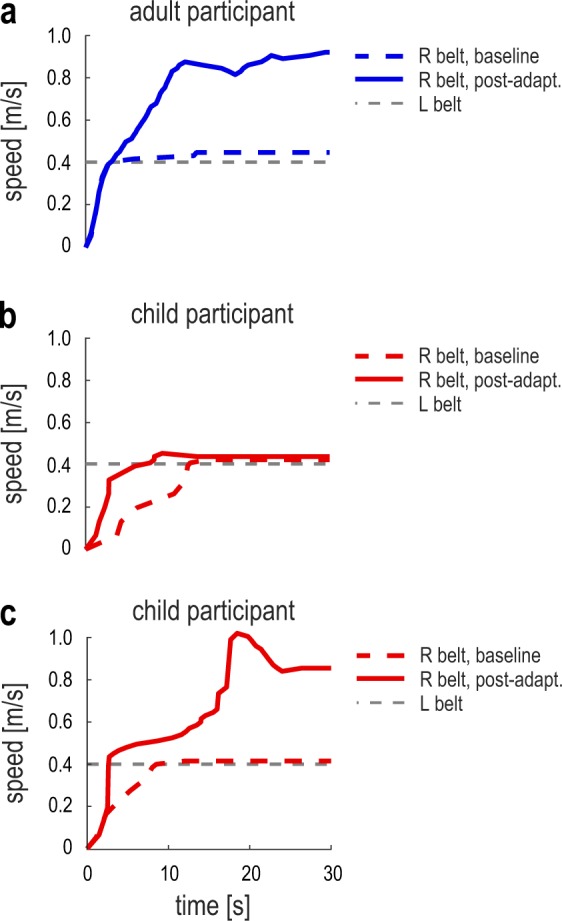
Individual participant examples (**a**: adult; **b,c**: children) of the treadmill belt speeds during the speed matching task. The gray dotted lines represent the speed of the left belt (constant at 0.4 m/s). The colored (blue or red) dotted lines represent the participant-controlled speed of the right belt during a baseline iteration of the task. The colored (blue or red) solid lines depict the speed of the right belt during the first post-adaptation iteration of the task. The adult and child participants in panels a and c overshot the speed of the right belt when matching it to that of the left, indicating that they recalibrated leg speed perception. The child participant in panel b had accurate perception of leg speed, indicating they did not recalibrate perception with adaptation.

Figure 4 shows examples of individual participant responses during two trials of the speed matching task (baseline and post-adaptation; these are the same participants shown in Fig. 2). In the baseline task (dashed line), the adult and children could accurately match the speed of the right leg to that of the left leg, indicating accurate perception of leg speed. The first post-adaptation task (solid line) was performed immediately after the participants walked for 15 minutes with split-belts (right belt 3 times faster than left). After adaptation the adult participant (panel a) demonstrated a robust overshooting in the right belt speed compared to the left. In other words, perception of leg speed was recalibrated such that the right leg (the leg that moved faster during adaptation) felt slower after adaptation. On the other hand, the child participant depicted in panel b was accurate in matching the speed of the belts after adaptation, indicating the child had no perceptual recalibration. The child participant depicted in panel c performed similar to the adult participant in the same task, indicating the child had adult-like perceptual recalibration. Hence, children varied in their ability to recalibrate leg speed perception with adaptation.

**Figure 5:**
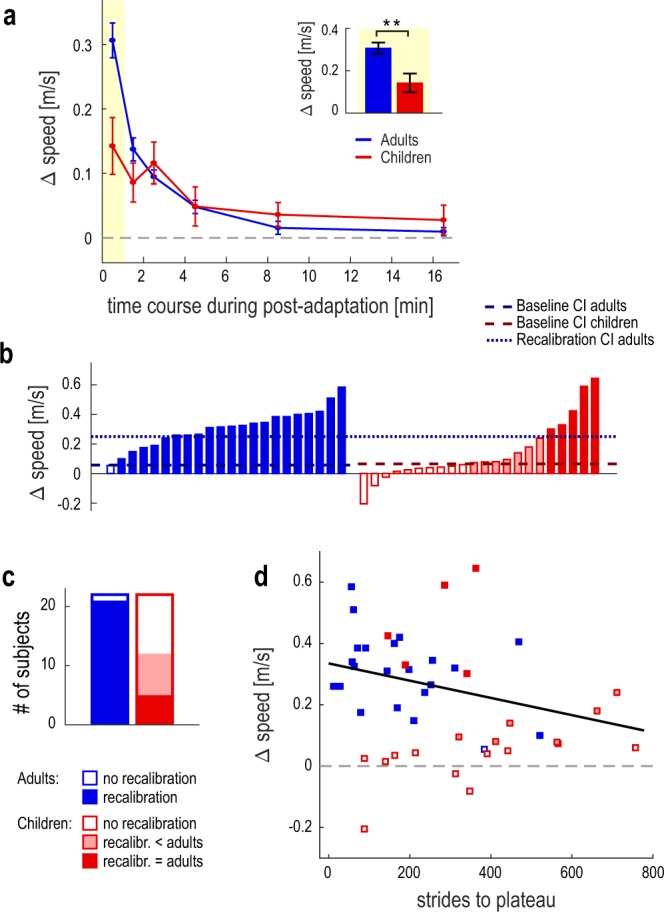
(**a**) Group data (mean ± SE) representing the perceptual recalibration measured by the six post-adaptation iterations of the speed matching tasks (see Methods). The dotted gray line represents a recalibration of zero. The inset of panel a shows the magnitude of the recalibration, captured by the first post-adaptation data point (highlighted). Error bars represent group mean ± SE. The recalibration was larger in adults than in children; significant difference between the groups is indicated by **(p < 0.01). (**b**) Magnitude of the perceptual recalibration for each participant. The dashed blue and red lines represent the threshold of recalibration for the adult and child groups respectively (baseline 95% CI, see Methods), and the dotted blue line depicts the threshold of adult-like perceptual recalibration (recalibration 95% CI, see Methods). Based on these confidence intervals, the colored and white areas of the bars correspond respectively to participants who did vs. did not change perception of leg speed with adaptation. For the child participants, the solid red area of the bar indicates participants who recalibrated perception to the same extent of adults, and the shaded area corresponds to participants who recalibrated less than adults (see Methods). (**c**) Proportion of participants in each group that showed perceptual recalibration. The color of the bars corresponds to the representation in Panel (b). (**d**) Perceptual recalibration versus strides to plateau for each adult and child participant; the appearance of each data point corresponds to the representation in Panel (b).

We measured task performance as the difference between the right and left belt speed at the end of each task iteration. We first ensured that accuracy and precision of the leg speed perception during baseline were similar between the adult and child groups (accuracy: 0.04 ± 0.01 m/s vs. 0.04 ± 0.01 m/s, t_(42)_ = 0.118, p = 0.907; precision: 0.05 ± 0.01 m/s vs. 0.03 ± 0.01 m/s, t_(42)_ = 1.679, p = 0.102). Baseline performance was then obtained for each participant by averaging the three baseline iterations. For each post-adaptation task, we evaluated task performance relative to baseline performance. Group post-adaptation performance of the leg speed perception task is shown in Fig. 5a. For both groups, we observed a robust bias in leg speed perception in the first post-adaptation task, indicating both adults and children recalibrated leg speed perception as they walked on split-belts (t_(21)_ = 11.322, p < 0.001; t_(21)_ = 3.237, p = 0.004). We also observe that the perceptual recalibration decayed as participants washed out in the motor domain, and by the end of adaptation participants in both the adult and child groups performed similarly to baseline (t_(21)_ = 1.583, p = 0.128; t_(21)_ = 1.197, p = 0.245).

To compare the perceptual recalibration between groups, we used a two-way repeated-measures ANOVA with factors Group and Time. We found a significant effect of Time (F_(5,210)_ = 28.571, p < 0.001) and a significant Group X Time interaction (F_(5,210)_ = 6.427, p < 0.001). Post-hoc tests revealed that, in the first post-adaptation time-point (inset of Fig. 5a), child participants demonstrated significantly less perceptual recalibration compared with adults (mean ± SE: 0.14 ± 0.04 vs. 0.31 ± 0.03 m/s; p = 0.003). Thus, although both children and adults had a change in leg speed perception following split-belt treadmill walking, the magnitude of perceptual recalibration was significantly smaller in children. This suggests that, although 6–8 years old children may have some degree of perceptual recalibration, this ability is not yet fully developed.

To explore this further, we wondered if individual child participants exhibited the reduced perceptual recalibration that we observed at group level, or if instead they exhibited a range of perceptual recalibration magnitudes. We present this analysis in Fig. 5b, which shows the magnitude of the perceptual recalibration (first post-adaptation time-point) for each participant. Importantly, children varied greatly in their ability to recalibrate perception: some children exhibited close-to-zero perceptual recalibration, while other children recalibrated perception to a similar extent as the adult group. The figure also shows that three children exhibited a negative perceptual recalibration: for two of the participants, this finding was explained by errors in the baseline speed matching tasks, conversely the other child selected a right speed that was slower than the left in the first post-adaptation speed matching task. White bars display the perceptual recalibration of participants who did not change their leg speed perception with adaptation. We determined that a participant did not change perception if their first post-adaptation measure of leg speed perception fell within the (one-sided) 95% confidence interval of the group baseline accuracy (see Methods). While only one adult participant did not recalibrate perception, 10 of the child participants did not show significant changes in leg speed perception. Solid red bars depict the recalibration of child participants who changed their leg speed perception to the same extent as the adult group (based on the 95% confidence interval of the first post-adaptation time-point in adults, see Methods). Of the 12 child participants who showed recalibration, only five recalibrated leg speed perception to adult level, while seven participants showed reduced recalibration. Figure 5c summarizes these findings, suggesting a transition between no recalibration and adult-like perceptual recalibration in 6–8 years old children.

### Are the motor and perceptual changes related?

Given that children adapt their motor pattern slower than adults and have a reduced perceptual recalibration, we wondered if there was a relationship between these phenomena. We performed post-hoc correlation analyses that included individual performance of all adults and children. We first looked at the relationship between the perceptual recalibration magnitude and the strides to plateau (one motor measure of adaptation rate), and we display this analysis in Fig. 5d. We found no correlation between the two measures (r = −0.276, p = 0.07), and we specifically observed that 2 of the 7 children who adapted at a similar rate to adults (strides to plateau < 245) also exhibited adult-like perceptual recalibration, but the other 5 children did not recalibrate perception. We additionally assessed the earliest change in step length asymmetry (taken as the average of the first 30 strides, representing the initial fast component of motor adaptation) and found that this did also not relate to the magnitude of the perceptual recalibration (r = −0.012, p = 0.938).

### Do children’s age or gender affect motor and perceptual recalibration?

**Figure 6:**
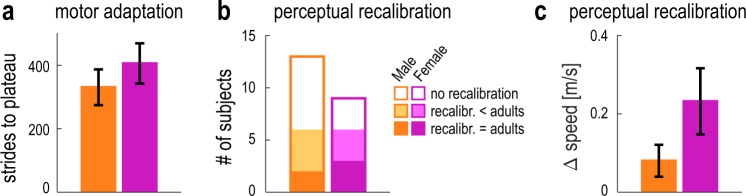
(**a**) Number of strides to reach plateau for male (orange) vs. female (pink) child participants. Error bars represent group mean ± SE. (**b**) Proportion of male and female child participants that showed perceptual recalibration. The white areas of the bars correspond to participants who did not change perception of leg speed with adaptation. The solid red areas of the bars indicate participants who recalibrated perception to the same extent of adults, and the shaded areas correspond to participants who recalibrated less than adults. (**c**) Magnitude of the perceptual recalibration for male (orange) vs. female (pink) child participants. Error bars represent group mean ± SE.

We finally wondered if we could see an effect of maturation on the motor adaptation and the perceptual recalibration within our child group. First, we investigated if there was an effect of age: Pearson correlations revealed that the strides to plateau measure was not correlated with the children’s age in months (r = −0.15, p = 0.52); similarly, the perceptual recalibration was also not correlated with the children’s age (r = 0.30, p = 0.17). We next investigated whether the motor adaptation and perceptual recalibration would follow a differential development in boys and girls, as we know that both the cerebrum and cerebellum mature earlier in girls^33,35^. In this exploratory analysis, we used non-parametric statistics to describe these data. We first ensured the age in months was comparable between the children in the female and male subgroups (mean ± SD: 88 ± 10 vs 92 ± 10 months; U = 45.500, p = 0.393). Girls and boys took a similar number of strides to reach their plateau step length asymmetry during adaptation (Fig. 6a, mean ± SE: 406 ± 63 vs 331 ± 56 strides; U = 47.000, p = 0.471), suggesting that gender did not affect the rate of motor learning. We next assessed how many of the female and male child participants recalibrated leg speed perception. Interestingly, 2/3 of the female child participants changed leg speed perception with adaptation (with respect to the child group baseline, see Methods), while less than half of the male child participants showed any recalibration (Fig. 6b). The magnitude of the perceptual recalibration (i.e. the result from the first post-adaptation speed matching task) also appeared larger in girls with respect to boys (Fig. 6a, 0.23 ± 0.08 vs 0.08 ± 0.04 m/s), but the difference was not significant (U = 36.000, p = 0.144). Importantly, we saw no effect of gender in our adult group. Female and males took a similar number of strides to reach plateau (mean ± SE: 169 ± 40 vs 197 ± 48 strides; U = 52.000, p = 0.628) and recalibrated perception to a similar amount (mean ± SE: 0.31 ± 0.04 vs 0.30 ± 0.04 m/s; U = 58.000, p = 0.923).

## Discussion

Motor learning in humans is a complex phenomenon, that relies on multiple mechanisms working together to enable us to move in constantly changing environments^1,3,5,6^. We modify and learn new movements every day, yet much is still unknown about the neural circuits that underlie motor learning. Our study provides new information regarding the development, in childhood, of the processes underlying a form of motor learning called adaptation. Specifically, we studied locomotor adaptation in 6–8 years old children and adults using a split-belt treadmill walking paradigm, which has been previously shown to induce changes in both gait pattern and perception of leg speed in adults^4,23,24^. We demonstrated that the locomotor-adaptation-driven changes in both movement and perception are not fully mature in 6–8 years old children, as child participants adapted slower and recalibrated their movement perception less than adults. We suggest that, in the 6–8 years age window, some of the neural mechanisms of adaptation are just beginning to come online, explaining the underdeveloped recalibration of movement and perception that we observed in children.

Consistent with previous work^20^, we found that children aged 6–8 years learned the new gait pattern with split-belt adaptation to the same degree as adults, but they learned more slowly. The finding that children adapt slower than adults is consistent with studies across different types of movements^18–20^. Adults normally display a characteristic double exponential time-course of adaptation, with an initial rapid learning phase followed by a slower phase^15,20^. This pattern has been described using a state-space model, and was hypothesized to represent two distinct mechanisms of learning, a “fast process” and a “slow process”, that may have distinct neural underpinnings^15^. During split-belt walking, young children do not show this double exponential learning pattern. Instead, children under ~age 9 display a pattern that is well described by a linear or single exponential function^20^. It has been suggested that the neural mechanism underlying the fast process of adaptation may reach maturation only after age 9, explaining why the time-course of adaptation in younger children is linear instead of double exponential^20^. Consistent with this hypothesis, here we observed a transition in the time-course of adaptation in the 6–8 years old age window, with some children adapting slower than adults (with the time-course of learning appearing linear), and other children adapting at a rate comparable to adults (with the time-course of learning appearing exponential).

In addition to the slower time-course of motor learning, 6–8 years old children exhibited significantly less recalibration of leg speed perception. Perceptual recalibration in adults accounted for ~40% of the actual leg speed difference (i.e., by the end of adaptation adult participants perceived their leg speeds to be only 0.49 m/s different when in reality the difference was 0.8 m/s). On average, children recalibrated perception to ~20% of the speed difference (i.e., they perceived a 0.66 m/s difference in leg speed, more similar to the true value of 0.8 m/s). Interestingly, not all of the child participants had reduced perceptual recalibration. Rather, they ranged from having no perceptual recalibration to having adult-like recalibration, suggesting that the ability to recalibrate leg speed perception with adaptation is just beginning to come online in the 6–8 years age window.

One hypothesis that would explain the differences in both motor and perceptual domains between children 6–8 years of age and adults, is that the perceptual recalibration and the fast process of adaptation rely on the same neural mechanism, which starts to come online during this developmental window. Several recent studies have worked towards understanding the relationship between the motor and perceptual recalibration processes that occur with adaptation^28,36,37^. ‘t Hart and Henriques 2016 suggested that the perceptual recalibration may be a marker of one of the components contributing to motor adaptation. Specifically, changes in movement perception may be indicative of the recalibration of our predictive internal models, which associate our motor commands with their expected perceptual consequences. Data from several groups suggest that internal models are not yet fully developed in school-aged children^38–40^, especially for complex tasks requiring balance^41^, such as walking. More specifically, the association between an action and its sensory consequences may not be recalibrated as flexibly in children as in adults^42^. Therefore, immaturity in these predictive models of movement could explain both the slower rate of error correction of the movement pattern and the smaller change in movement perception with adaptation in our child versus adult group.

On the other hand, it is also possible that children’s slower adaptation and reduced perceptual recalibration are markers of somewhat separate neural processes. Studies in both reaching^36^ and walking^37^ adaptation have suggested a degree of independence between these two phenomena. In reaching, proprioceptive recalibration has been shown to be slower than motor adaptation^36^. In walking, our group has shown that adults adapting repeatedly on the split-belt treadmill over days leads to different savings patterns in the motor versus perceptual domains^37^. Consistent with this hypothesis, we were unable to find correlation between measures of adaptation rate and magnitude of perceptual recalibration. Together, these findings suggest that there is not a one-to-one relationship between motor and perceptual recalibration. It is possible that perceptual recalibration may be related to some, but not all, of the processes underlying motor adaptation, however our data cannot address this hypothesis, as we do not have separate measures of the fast process and slow process for each child. We were unable to accurately obtain these measures because model fits are much more challenging for individual children, as compared to adults, due to high stride-to-stride variability. This is a limitation of our study, as our analysis could not fully address the question of whether the development of the fast process of adaptation and the perceptual recalibration are related.

Hence, differences between children and adults in the motor and perceptual domains of adaptation could be explained by immaturity in either the same, or different neural mechanisms. Several sensorimotor areas are undergoing development in 6–8 years old children^31–33^. Development of both phenomena could be related to the ongoing maturation of cortical motor, sensory, and frontal areas^31,32^. Maturation of cerebral cortex begins in primary sensory and motor areas in early childhood, and continues in areas associated with higher order integration and processing (such as prefrontal cortex and parietal cortex) until late adolescence^31,32^. As such, immaturity in different cerebral networks may contribute to the motor and perceptual phenomena studied here.

Given the prolonged cerebellar development in childhood^33^, another interpretation of our findings is that both motor and perceptual recalibration may be related to the recalibration of an internal predictive model thought to be housed in the cerebellum^26–28^. There is evidence from patient and animal studies, across several movement types, to support the involvement of the cerebellum in motor adaptation^9–13,20,43^, as well as perception and perceptual recalibration during self-generated motion^25–27,44–46^. In the context of split-belt walking, we specifically find that children are similar to cerebellar patients in both the motor and perceptual behavior, suggesting that cerebellar development could explain the immaturity of both motor and perceptual recalibration in 6–8 years old children^20,25^. First, Vasudevan *et al*.^20^ showed that young children and patients with cerebellar ataxia have remarkably similar aftereffects across multiple gait parameters following locomotor adaptation. Further, Statton *et al*.^24^ showed that patients with cerebellar ataxia have, on average, reduced perceptual recalibration following locomotor adaptation compared to healthy adults, and that ataxia severity is a strong predictor of whether a person recalibrates perception. Similar to our findings in 6–8 years old children, cerebellar patients were impaired in both the motor and perceptual domains, though no correlation was found between the magnitude of motor and perceptual learning. Finally, the cerebellum develops more slowly in boys than girls, which could underlie why fewer boys than girls recalibrated leg speed perception^33^.

In summary, it is possible that differences in the motor and perceptual components of adaptation in children versus adults are due to immaturity in different neural circuits, though a more parsimonious hypothesis would be that the development of the same predictive mechanisms may explain the differences in both domains. The prevailing theory is that the cerebellum is involved in hosting and recalibrating our internal predictive models of movement^12,47^, and this recalibration may be involved in changing both movement and perception during adaptation^27,28,48,49^. Hence, less mature cerebellar circuitry that mediates changes to our internal models of movement may explain both the slower rate of locomotor adaptation and the reduced recalibration of leg speed perception observed in 6–8 years old children. However, our study could not provide evidence for or against a direct relationship between perceptual recalibration and the fast process of adaptation. Future work is needed to ascertain how motor adaptation and perceptual recalibration are related. Ultimately, understanding the neural circuits that underlie different forms of motor learning is essential for developing targeted rehabilitation interventions for different patient populations.

## Materials and Methods

### Participants

22 healthy children (age range 6–8 years old, 9 females) and 22 healthy adults (18–29 years old, 12 females) participated in this study. All participants were screened for self-reported cardiovascular, neurological or motor dysfunctions. Written informed consent was provided by participants (adults) or legal guardians (children) before participation and the experimental protocol was approved by the Johns Hopkins Institutional Review Board. The experimental methods were carried out in accordance with the relevant guidelines and regulations.

### Experimental setup and design

#### Split-belt walking

All participants walked on a custom-built treadmill (Woodway USA, Waukesha, WI) with two separate belts driven by independent motors. The belts can be driven to move at the same speed (“tied-belt”) or at different speeds (“split-belt”). Speed commands for each belt were sent to the treadmill through a custom Python program in the Vizard (WorldViz) development environment. Participants were positioned in the middle of the treadmill with one leg on each belt, separated by a divider to avoid tripping. They wore a safety harness which did not provide any body-weight support, and held onto a handrail in front of them. The handrail was at elbow level for adults and at chest level for children. All children wore a safety clip tethered to an emergency stop switch on the treadmill. A large television screen was placed in front of the treadmill, which was used to provide simple visual feedback during some phases of the experiment (see below, *Speed matching task*).

#### Experimental paradigm

The paradigm (Fig. 1a) began with a 3-minute baseline phase, where the treadmill belts were tied at 0.4 m/s. All participants then walked for 15 minutes with split-belts (left belt moving at 0.4 m/s and right belt moving at 1.2 m/s) in the adaptation phase. Participants experienced a 10-second catch trial (belts tied at 0.4 m/s) after 10 minutes of adaptation, then resumed split-belt walking for the remaining 5 minutes of adaptation. During post-adaptation, belts were tied at 0.4 m/s for a total of 13.5 minutes of walking.

To assess participants’ leg speed perception during baseline, we performed three iterations of the speed matching task (see below) after 1, 2, and 3 minutes of tied-belt walking. To measure the perceptual recalibration following split-belt walking, six iterations of the speed matching task were performed in the post-adaptation phase, specifically 0, 1, 2, 4, 8, and 16 minutes after the end of adaptation.

#### Speed matching task

To measure leg speed perception, we used a speed matching task adapted from Vazquez *et al*.^24^ and based on the psychophysical method of adjustment. A custom Python program was used to collect input from participants to update the right treadmill belt speed during walking and provide feedback of task timing. Specifically, participants were positioned on the treadmill and instructed to place their left hand on a handrail in front of the treadmill and their right hand on a small keypad (Fig. 1b). The left leg was driven to walk at a constant speed of 0.4 m/s, while the right leg was initially not moving. Participants were instructed to press up or down arrows on the keypad in front of them to adjust the speed of the right leg until they perceived it to match the speed of the left leg. Participants were given 30 s to complete the task and were provided feedback on the amount of time remaining via a television monitor in front of the treadmill. Vision of the legs was obstructed via an opaque drape, and auditory cues of speed from the treadmill motors were canceled via headphones playing white noise.

Note that the key presses did not always change the right belt speed by the same increment. This was done so that participants could not simply memorize the number of key presses needed for the task. When the right leg was within the range 0.0–0.35 m/s, ‘up’ key presses resulted in speed increments of either 0.05, 0.055, or 0.065 m/s. These increments were varied with each iteration of the task. Once the speed passed 0.35 m/s, key presses resulted in a smaller change in speed, 0.015 m/s, to allow for fine control of speed as the right leg approached the target speed. ‘Down’ keypresses always resulted in a speed decrement of 0.015 m/s. Prior to the start of the experiment, child participants were provided a demonstration of the speed matching task, and some of the children briefly practiced using the keypad to change the right belt speed.

### Data collection and analysis

#### Optotrak motion analysis

Kinematic data sampled at 100 Hz were collected during walking using Optotrak (Northern Digital, Waterloo, ON, Canada). Infrared-emitting markers were placed bilaterally on the toe (fifth metatarsal head), ankle (lateral malleolus), knee (lateral femoral epicondyle), hip (greater trochanter), pelvis (iliac crest), and shoulder (acromion process).

#### Motor measures

Kinematic data were analyzed using Matlab (The MathWorks, Natick, MA). The primary outcome for motor adaptation was step length asymmetry:

1 $$Step\,length\,asymmetry=\frac{S{L}_{f}-S{L}_{s}}{S{L}_{f}+S{L}_{s}}$$

where SL_f_ and SL_s_ represent the step length of the fast leg and slow leg, respectively. Step length was calculated as the anteroposterior distance between the malleolus markers of each leg at heel strike. A step length asymmetry of 0 indicates symmetrical walking.

We quantified the rate of adaptation of step length asymmetry for each participant as the number of strides needed to reach plateau^34^. Data from each participant were pre-smoothed using a 5-point moving average. We defined the plateau range for each participant as the mean ± SD of the last 30 strides of adaptation, and we then analyzed the adaptation data to find the number of strides until five consecutive strides fell within the participant’s plateau range. For each child participant, we also assessed whether their rate of adaptation was comparable to that of the adult group. Specifically, we first calculated the 95% one-sided lower confidence interval of the adult group stride to plateau measure, and we then assessed whether the stride to plateau measure of each child participant fell within the adult range.

#### Perception measures

We recorded changes in the right belt speed as the speed matching task was performed, and defined task performance as the difference between right and left belt speed at the end of each 30 second trial. We first compared baseline performance between the groups. For each participant, we quantified the baseline accuracy as the magnitude of the average baseline performance over the three speed matching tasks (*accuracy* = *abs (mean over three tasks (Final R Speed – Final L Speed))*). For each participant we additionally quantified baseline precision as the standard deviation of the baseline tasks performance (*precision* = *std over three tasks (Final R Speed – Final L Speed)*).

We then assessed how leg speed perception changed following adaptation. For reference, we calculated the average baseline performance by taking the average of each participant’s three baseline trials. We then evaluated post-adaptation performance relative to baseline (i.e. Δ speed). For each of the 6 post-adaptation trials, a value of 0 thus indicates that the participant had the same perception of leg speed as at baseline, whereas positive values indicate that the participant perceived the right belt to be moving slower than they had at baseline.

We also quantified if each individual participant showed significant changes in leg speed perception following adaptation. We first calculated the 95% one-sided lower confidence interval of the baseline speed matching tasks accuracy for the child and adult groups. We then considered a participant to have a significant perceptual recalibration if their performance during the first post-adaptation speed matching task fell outside the confidence interval of their group’s baseline accuracy. For child participants who showed significant changes in leg speed perception, we further assessed whether their perceptual recalibration was comparable to that of adult participants. Specifically, we first calculated the 95% one-sided upper confidence interval of the adult group recalibration (performance during the first post-adaptation task). We then assessed whether the recalibration of each child participant fell within the adult range.

Finally, we were interested in assessing whether there were gender-related differences in the motor adaptation or perceptual recalibration. We therefore compared the following measures between female and male participants in each group: strides to plateau, magnitude of the perceptual recalibration, and number of participants that recalibrated perception (child group only).

### Statistical analysis

#### Motor measures

Mean step length asymmetry for the adult and child groups was calculated at distinct time epochs during the experiment: baseline (mean of all strides), initial adaptation perturbation (first 5 strides of adaptation), catch trial (first 3 strides of catch trial) and adaptation plateau (last 30 strides).

We first compared baseline step length asymmetry between the groups using an independent sample t-test. We then used a repeated-measures ANOVA to assess changes in step length asymmetry between adult and child groups across epochs: initial adaptation perturbation, catch trial and adaptation plateau. The Greenhouse-Geisser correction was used as needed to correct for violations of Mauchly’s test of sphericity.

To evaluate the rate of motor adaptation, an independent sample t-test was used to compare the number of strides to reach the plateau range between children and adults. Additionally, we used a Mann-Whitney U test to compare the strides to plateau between female and male participants in each group.

#### Perception measures

First, we used independent sample t-tests to compare the accuracy and precision of the baseline performance between the child and adult groups. We also used independent sample t-tests to compare the magnitude of perceptual recalibration to zero, for the first and last post-adaptation data points, for each group separately. We next compared the magnitude of perceptual recalibration (post-adaptation task performance relative to baseline, i.e. Δ speed) for the six post-adaptation time points between groups, using two-way repeated measures ANOVA with factors Group and Time. The Greenhouse-Geisser correction was used as needed to correct for violations of Mauchly’s test of sphericity.

We additionally used the Pearson Correlation Coefficient to investigate the relationship between children’s age in months, and the perceptual recalibration and stride to plateau measures (child group only). Using the data from both groups we also looked at the correlation between the perceptual recalibration and the following measures of motor adaptation: strides to plateau, and the average step length asymmetry over the first 30 strides of adaptation. We finally compared the first post-adaptation time point between female and male participants in each group using a Mann-Whitney U test. To ensure any effect in the child group was not due to age differences, we also used a Mann-Whitney U test to compare the age in months between boys and girls.

SPSS (IBM) was used for all statistical analysis and the α level was set at p = 0.05. When applicable, all post-hoc tests were performed with the Bonferroni correction.

## Data Availability

The datasets analyzed during the current study are available from the corresponding author on reasonable request.
